# Application of response surface methodology for optimization of metal–organic framework based pipette-tip solid phase extraction of organic dyes from seawater and their determination with HPLC

**DOI:** 10.1186/s13065-019-0572-0

**Published:** 2019-04-23

**Authors:** Sayyed Hossein Hashemi, Massoud Kaykhaii, Ahmad Jamali Keikha, Elahe Mirmoradzehi, Ghasem Sargazi

**Affiliations:** 1 Department of Marine Chemistry, Faculty of Marine Science, Chabahar Maritime University, Chabahar, Iran; 2 Department of Chemistry, Faculty of Sciences, University of Sistan and Baluchestan, Zahedan, 98155-674 Iran; 3 Department of Mechanical Engineering, Faculty of Marine Engineering, Chabahar Maritime University, Chabahar, Iran; 4 Department of Nano Chemistry, Graduate University of Advanced Technology, Kerman, Iran

**Keywords:** Azo dyes, Metal–organic framework, Pipette tip solid phase extraction, Response surface methodology, Box–Behnken design, Seawater analysis

## Abstract

This paper describes the application of response surface methodology (RSM) to develop a miniaturized metal organic framework based pipette-tip solid phase extraction for the extraction of malachite green (MG), rhodamine B (RB), methyl orange (MO) and acid red 18 (AR) dyes from seawater samples and their determination by high performance liquid chromatography. The effects of various parameters such as pH of the sample solution, type and amount of added salt, type and volume of eluent solvent, concentration of surfactant (triton X-114), sample volume, and number of cycles of extraction and desorption were investigated and optimized by two methods of one-variable-at-a-time and RSM based on Box–Behnken design. Under optimum conditions, the linear range of the method was 0.5–200.0 µg/L for RB and MG and 1.0–150.0 µg/L for AR and MO. Limits of detection of the analytes were obtained in the range of 0.09–0.38 µg/L. Reproducibility of the method (as RSD %) was better than 6.4%. The method has been successfully used for analysis of four dyes in seawater of Chabahar Bay.

**Electronic supplementary material:**

The online version of this article (10.1186/s13065-019-0572-0) contains supplementary material, which is available to authorized users.

## Introduction

**Fig. 1:**
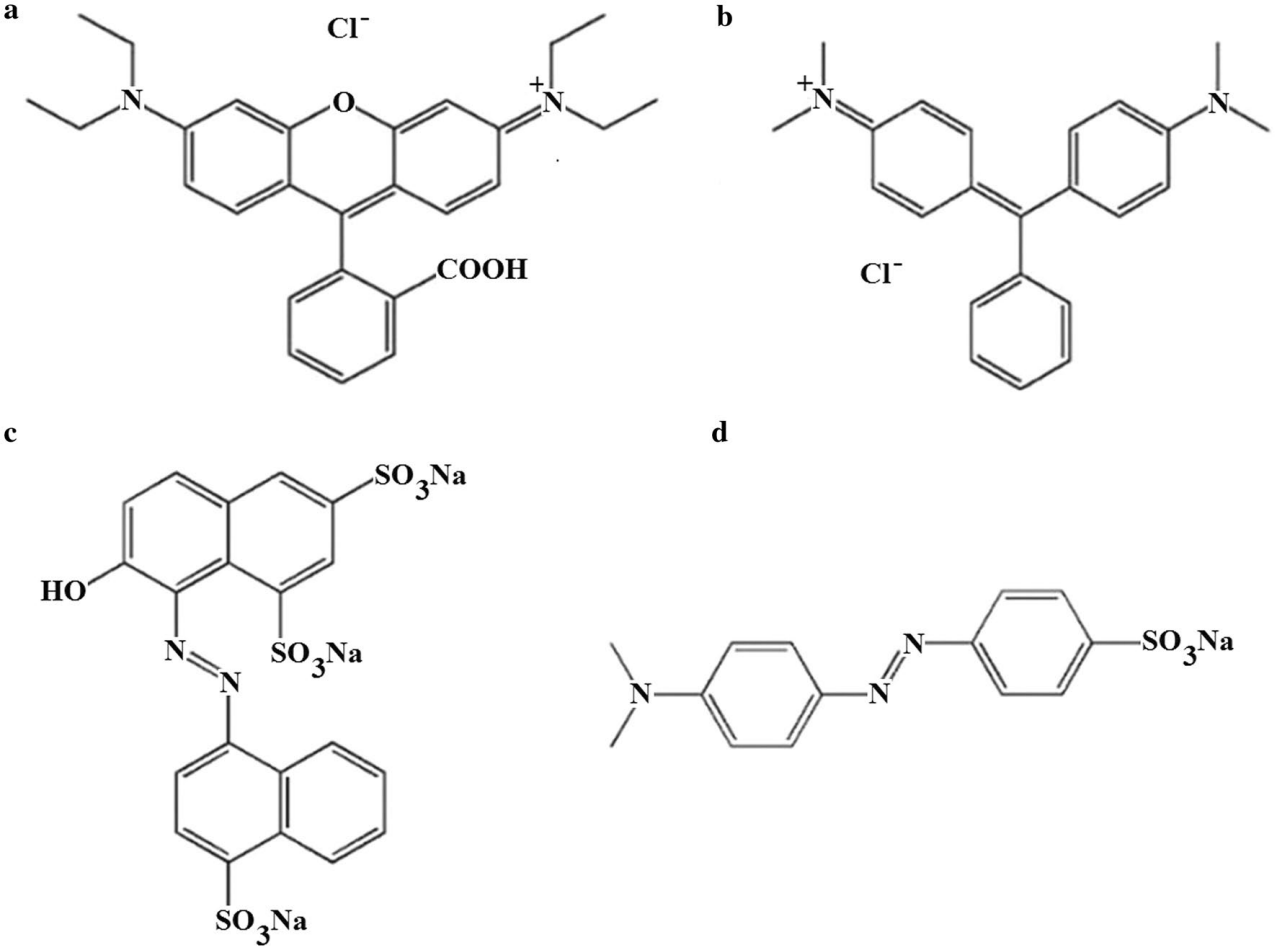
Structure of dyes studied in this paper **a** rhodamine B, **b** malachite green, **c** acid red 18, **d** methyl orange

Rhodamine B (RB) (Fig. 1a), is among the oldest and most commonly used synthetic dyes that have been recently identified as possible illegal additives in foods exported from European Union and China [1]. It belongs to the class of xanthenes dyes, a basic red cationic dye that is highly soluble in water, methanol and ethanol. This dye is used widely as a colorant in textiles and plastic industries. RB is harmful if swallowed with human beings and cause irritation to the skin, eyes and respiratory tract. Also, it has been shown to have carcinogenicity, reproductive and developmental toxicity, neurotoxicity and chronic toxicity towards human and animals [2]. Malachite green (MG, Fig. 1b), although a forbidden dye, has been widely applied illegally as a fungicide and parasitical and in the fish industry as an antimicrobial, antiseptic and ectoparasitic agent, because of its high efficiency and low cost [3–5]. Acid red 18 (AR, Fig. 1c), is a popular food color, not toxic but can be harmful if used in excess [6, 7]. Methyl orange (MO, Fig. 1d), have many application as textile dyeing stuff and staining agents in laboratories [8]. These dyes are of the most abundant applied dying agents throughout the world and therefore can find their way to the environmental sources such as seawater as hazardous pollutants [9, 10].

Different techniques such as liquid chromatography–mass spectrometry (LC–MS) [11, 12], liquid chromatography–tandem mass spectrometry (LC–MS/MS) [13], gas chromatography–mass spectrometry (GC–MS) [14], capillary electrophoresis [14], high performance liquid chromatography (HPLC) [14, 15], high performance liquid chromatography–mass spectrometry (HPLC–MS) [16] and spectrophotometry [8, 17] have been used for determination of dyes in complex samples. Each of these techniques has disadvantages. Spectrophotometry lacks the required selectivity and sensitivity, while LC–MS, LC–MS/MS, GC–MS and HPLC–MS are relatively expensive techniques and capillary electrophoresis is slow for the determination of analytes.

Use of an enrichment step for determination of dyes is normally required. This is mainly due to their low concentration or the severe matrix interference in real samples such as seawater [18–20]. Several extraction methods such as liquid–liquid extraction (LLE) [21], liquid phase microextraction (LPME) [22], solid phase extraction (SPE) [23], solid phase microextraction [24], molecular imprinted polymer (MIP) [25], cloud point extraction (CPE) [26] and micro-cloud point extraction [8, 17] have been developed to determine organic dyes in different matrices.

Metal–organic frameworks (MOFs) are three dimensional crystalline porous materials having different geometries and functional groups within the channels/cavities, which are synthesized using mixing organic linkers and metal salts, often under hydrothermal or solvothermal conditions. The unique characteristic of the hybrid solids are adjustable pore-sizes and controllable structural properties, extra ordinarily large porosity, low density and their very high surface areas. MOFs have been considered as promising candidate materials for different applications including adsorption, removal, separation, selective extraction and pre- concentration of various analytes [27, 28].

Pipette-tip solid phase extraction (PT-SPE) is a representative SPE technique because of its miniature device and use of reduced amount of reagents and less time consumption [29]. For PT-SPE, an ordinary pipette tip acts as the extracting column, packed with sorbent. This technique has been successfully used in many applications [30, 31].

Response surface methodology (RSM) can be summarized as a compilation of statistical tools and method for constructing and exploring estimated function relationship between a response variable and set of design variable. It is the collection of mathematical and numerical methods that are suitable for modeling and analysis of the problems having numerous variables influencing the response, and objective is to optimize the response. The most extensive application of RSM can be found in industrial world, where a number of input variables affect some performance measures, called the response, in ways which are not easy or unfeasible to depict by a rigorous mathematical formulation [32, 33].

In the present work, we synthesized a novel Co-MOF and used it for simple, fast and sensitive PT-SPE of RB, MG, AR and MO organic dyes in seawater samples and their determination with HPLC. Parameters affecting PT-SPE were optimized by two methods of one variable-at-a-time and RSM, based on Box–Behnken design. This is the first report on using Co-MOF for pipette-tip solid phase extraction of dyes in Chabahar Bay (Oman Sea).

## Experimental

### Apparatus

A Knauer HPLC (Germany) equipped with a EA4300F smart line pump and a smart line auto sampler 3950, was used for all analyzes. Detection system was a diode array spectrophotometer, used at wavelengths of 448 nm for MO, 510 nm for AR, 555 nm for RB and 618 nm for MG. Analytical column was a 250 × 4.6 mm Eurospher 100-5 C_18_ utilizing the same pre-column. ChromGate V3.1.7 software was used for chromatographic data handling. The injection loop volume was 20 µL. A model 630 Metrohm (Switzerland) pH meter was employed for pH determination.

### Reagents

All dyes and chemical reagents were of analytical grade and were purchased from Merck KGaA (Darmstadt, Germany). The HPLC grade methanol, acetonitrile and water were also obtained from the same company. Milli-Q^®^ water (18.3 MʹΩ/cm) was used throughout the run after filtering through 0.22 mm Nylon membrane. Triton X-114 (5% v/v) solutions was prepared at 70:30 (v/v) water/methanol and used as the surfactant. Stock solution of each dye with a concentration of 500 mg/L was prepared with dissolving of 0.0500 g of each dye in distilled water in 100 mL flasks. Working solutions were prepared daily by proper dilution of stock solutions.

### Synthesize and characterization of Co-MOF adsorbent

**Fig. 2:**
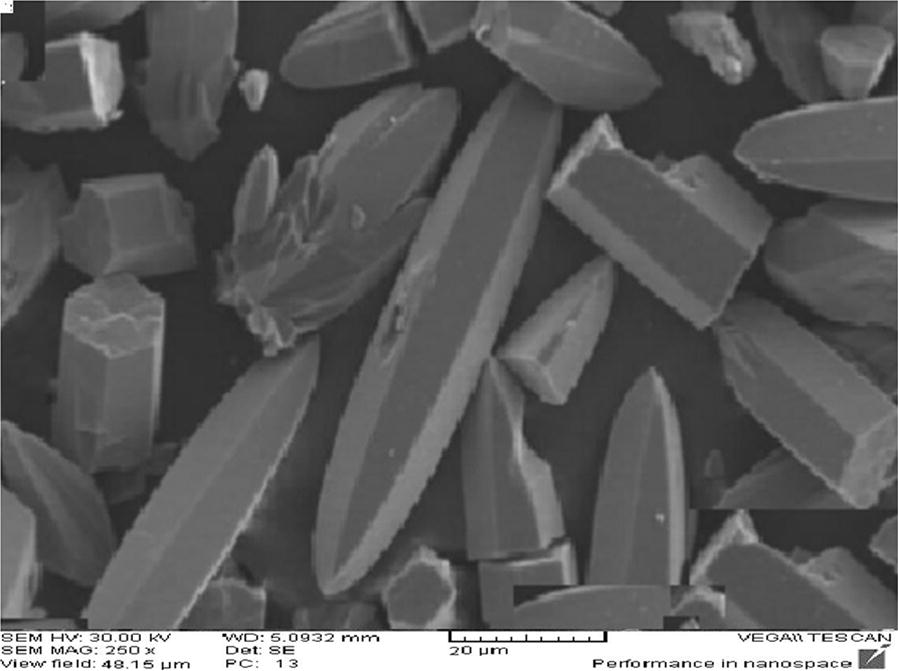
Scanning electron microscopy image of the synthesized Co-MOF

Synthesize of Co-MOF was according to the work of Sargazi et al. [34]. Briefly, 5.62 mmol of cobalt nitrate and 1.85 mmol of pyridine 2, 6-dicarboxylic acid were dissolved in 14 mL of ethanol. Obtained solution was transferred into a Teflon reactor with a tight cap and kept for 7 h at 85 °C. The product was washed with dimethylformamide. After mixing and dissolving the reactants, the clear solution radiated in the ultrasound bath for 13 min at working condition of 160 W, 1 kJ, and 21 kHz. Synthesized adsorbent was stored in 4 °C. Scanning electron microscopy (Fig. 2) showed an average size of 17 µm for synthesized MOF. By BET (Brunauer, Emmett and Teller), the specific surface area of Co-MOF was determined 3000 m^2^/g.

### Pipette-tip solid phase extraction procedure

PT-SPE of dyes was performed using an Extra GENE tip mounted on a variable 150 µL volume pipettor (Dragon Labs, USA). 8 mL of an aliquot of sample solution containing appropriate amounts of dye was transferred into a 10 mL flask and proper amount of triton X-114 (0.15% v/v for MG and AR and 0.20% v/v for RB and MO) and 150 mg of KCl was added. Then pH of solutions were adjusted to the desired value (pH = 3.0 for MG and RB, 6.0 for AR and 6.9 for MO) with drop-wise addition of either 1 mol/L of HCl or 1 mol/L of NaOH. PT-SPE carried out by loading the sample solution into the cartridge and washing out with 0.5 mL of methanol–water (1:1). After the analyte retained on the MOF sorbent, it was eluted using 300 µL (for RB, MO and AR) and 250 µL (for MG), of methanol contain 5% acetic acid. Finally eluted solvent was filtered through a 0.45 µm filter and was injected into HPLC for analysis.

## Results and discussion

### Chromatographic conditions

Various mobile phases were investigated consisting of methanol, acetonitrile and water in different combinations and pH settings. Finally a gradient of 85% B at 0–3.5 min and 100% B at 3.5–10 min was selected; in which eluent A was water and eluent B was acetonitrile which was adjusted to the pH 5.25 using acetic acid at a flow rate of 0.8 mL/min. The column oven temperature was maintained at room temperature and the mobile phase was degassed using a stream of helium prior to use.

### Optimization of MOF-PT-SPE

In order to achieve the best efficiency of the MOF-PT-SPE, different factors affecting extraction efficiency were optimized using two methods of one-variable-at-a-time and RSM based on Box–Behnken design. A standard aqueous solution at concentration of 150.0 µg/L for AR and MO and 250 µg/L for RB and MG was used for optimization experiments. Each experiment repeated at least three times.

#### Effect of type of the eluent solvent

Different solvents as eluent were studied for elution of dyes from the MOF sorbent, including methanol, methanol/acetic acid (1:2), methanol/acetic acid (1:1), methanol/acetic acid (2:1), methanol containing 5% acetic acid, acetonitrile, ethanol, methanol/H_2_O (1:1), H_2_O, acetone and acetic acid. Methanol containing 5% acetic acid showed the best efficiency for all analytes.

#### Effect of amount of sorbent

The effect of amount of MOF for preconcentration and determination of selected dyes in pipette tip was investigated in the range 1.0–2.5 mg. The results showed that the percent of extraction increases to 2.0 mg of MOF and then the recovery decreases. So, 2.0 mg of sorbent in pipette tip was used for further experiments.

#### Effect of type and amount of salt

To investigate effect of type and amount of salt on extraction efficiency of dyes, NaCl, KCl and Na_2_SO_4_ as common salts were selected and used for MOF-PT-SPE of dyes. Among them, KCl improved the extraction better than the other salts and hence selected as spiked salt in further works. To study effect of amount of KCl on extraction efficiency, various brine sample solutions containing different quantity of KCl in the range of 25–200 mg were prepared. The results indicated that the extraction efficiency of dyes is quantitative for amount of KCl greater than 200 mg. Hence, next runs were performed with saturation of the samples using 200 mg of KCl.

#### Effect of concentration of triton X-114

The concentration of triton X-114 as surfactant can effect on the extraction efficiency of dyes by MOF-PT-SPE; so, we tried to optimize its concentration. We found that by increasing the concentration of the surfactant, the extraction efficiency was also increases, but in the amounts more than 0.20 (for RB and MO) and 0.15 (for MG and AR) %v/v of triton X-114, a decrease in the extraction efficiency of dyes was observed. This is probably due to the dilution of the analytes in larger volumes of the surfactant.

#### Box–Behnken design

Four factors in three levels were utilized to consider and optimize the process factors which potentially have an effect on the extraction efficiency of the analytes by MOF-PT-SPE. The investigated factors and input variable for four dyes were pH (X_1_ or A), eluent volume (µL) (X_2_ or B), number of extraction cycles (X_3_ or C), and number of eluent cycles (X_4_ or D). Table 1 shows the levels of these variable which were coded as − 1 (low), 0 (central point) and 1 (high). The design of real runs is given in Additional file 1: Table S1.

**Table 1:** Levels or variables chosen for the trials

	A	B	C	D
MG	2 (− 1)	200 (− 1)	7 (− 1)	7 (− 1)
	3 (0)	250 (0)	9 (0)	9 (0)
	4 (+ 1)	300 (+ 1)	11 (+ 1)	11 (+ 1)
MO	6 (− 1)	250 (− 1)	3 (− 1)	3 (− 1)
	7 (0)	300 (0)	5 (0)	5 (0)
	8 (+ 1)	350 (+ 1)	7 (+ 1)	7 (+ 1)
RB	2 (− 1)	250 (− 1)	5 (− 1)	5 (− 1)
	3 (0)	300 (0)	7 (0)	7 (0)
	4 (+ 1)	350 (+ 1)	9 (+ 1)	9 (+ 1)
AR	5 (− 1)	300 (− 1)	3 (− 1)	3 (− 1)
	6 (0)	250 (0)	5 (0)	5 (0)
	7 (+ 1)	350 (+ 1)	7 (+ 1)	7 (+ 1)

The following quadratic equation (Eq. 1) can be used to explain the behavior of the system:

1 $${\text{Y}} =\upbeta_{0} + \sum\upbeta_{\text{i}} {{\text{X}}}_{{\text{i}}} + \sum\upbeta_{{\text{ii}}} {{\text{X}}}_{{\text{ii}}} + \sum\upbeta_{\text{ij}} {{\text{X}}}_{{\text{i}}} {{\text{X}}}_{{\text{j}}} +\upvarepsilon$$

In Eq. 1, *Y* is output; i.e. is the response of HPLC, which is the dependent variable; *i* and *j* are the index numbers of the model; β_0_ is the free or offset term, called intercept term; X_1_, X_2_, …, X_k_ are coded independent variables; B_i_ is the first-order (linear) main effect,; B_ii_ is the quadratic (squared) effect; β_ij_ is the interaction effect; and ε is the random error which allows for description or uncertainties between predicted and determined values [35].

For 4 selected dyes, subsequent equations explain the relationship between the four variables and response of HPLC (output, Y):

For MG:

2 $$\begin{aligned} {\text{Y}} = {\text{Peak Area}} & = [\left( { - 7 5 6 8 4 600000} \right) + \left( { 7 4 70 2 40000 \times {\text{A}}} \right) + \left( { 2 1 8 6 9 3000 \times {\text{B}}} \right) + \left( { 4 4 2 2 7 10000 \times {\text{C}}} \right) \\ & \quad + \left( { 4 5 4 8 2 90000 \times {\text{D}}} \right){-}\left( { 1 90 2 7 20 \times {\text{A}} \times {\text{B}}} \right){-}\left( { 40 4 1 8 800 \times {\text{A}} \times {\text{C}}} \right){-}\left( { 2 5 9 6 7 400 \times {\text{A}} \times {\text{D}}} \right) \\ & \quad + \left( { 4 80 70 9\times {\text{B}} \times {\text{C}}} \right){-}\left( { 1 7 5 7 6 1 {\text{E}} \times {\text{B}} \times {\text{D}}} \right) + \left( { 1 2 4 7 4 500 \times {\text{C}} \times {\text{D}}} \right){-}\left( { 10 9 1 5 10000 \times {\text{A}}^{ 2} } \right) \\ & \quad {-}\left( { 4 3 6 6 50 \times {\text{B}}^{ 2} } \right){-}( 2 5 10 5 9000 \times {\text{C}}^{ 2} ){-}( 2 4 8 8 7 1000 \times {\text{D}}^{ 2} )]^{0. 5} \\ \end{aligned}$$

For MO:

3 $$\begin{aligned} {\text{Y}} & = {\text{Peak area}} = 1.0 \left[\left({1.33912\times 10^{-3}} \right){-}\left({2.53546\times 10^{-4} \times {\text{A}}} \right){-}\left({3.45465\times 10^{ - 6} \times {\text{B}}} \right) \right. \\ & \left. \quad {-}\left( { 3.20448 \times10^{ - 6} \times {\text{C}}} \right){-}\left( {1.68106\times 10^{-5} \times {\text{D}}} \right) + \left( {1.53607\times 10^{-8} \times {\text{A}} \times {\text{B}}} \right) \right. \\ & \left. \quad + \left( {6.59639\times 10^{-8} \times {\text{A}} \times {\text{C}}} \right){-}\left( {4.18401\times 10^{-8} \times {\text{A}} \times {\text{D}}} \right){-}\left( {5. 80433\times 10^{ - 10} \times {\text{B}} \times {\text{C}}} \right) \right.\\ & \left. \quad {-}\left( {1.43491 \times 10^{ - 9} \times {\text{B}} \times {\text{D}}} \right) + \left( {6.02261 \times 10^{-7} \times {\text{C}} \times {\text{D}}} \right) + \left( { 2.08619 \times 10^{-5} \times {\text{A}}^{ 2} } \right) \right. \\ & \left. \quad + \left( {5.65699 \times 10^{ - 9} \times {\text{B}}^{ 2} } \right){-}\left( {5.83979 \times 10^{-8} \times {\text{C}}^{ 2} } \right) + \left( {1.31577 \times 10^{ - 6} \times {\text{D}}^{ 2} } \right)\right]\end{aligned}$$

For RB:

4 $$\begin{aligned} {\text{Y}} = {\text{Peak Area}} & = - 1 4 8 5 3 40 + \left( { 1 50 1 2 9\times {\text{A}}} \right) + \left( { 7 1 7 9. 1 6 7\times {\text{B}}} \right) + \left( { 1 4 60 2 7\times {\text{C}}} \right) + \left( { 1 6 7 7 5. 5 5\times {\text{D}}} \right) \\ & \quad - \left( { 6 3 6. 3 5\times {\text{B}} \times {\text{C}}} \right) + \left( { 3 5. 5 2 5\times {\text{B}} \times {\text{D}}} \right){-}\left( { 2 4 8 5 3. 6 3 3 3 3\times {\text{A}}^{ 2} } \right){-}\left( { 1 1. 4 6 7 5 5\times {\text{B}}^{ 2} } \right) \\ & \quad {-}\left( { 5 9 5 4. 3 7 70 8\times {\text{C}}^{ 2} } \right){-}\left( { 1 8 1 4. 8 4 5 8 3\times {\text{D}}^{ 2} } \right) + \left( {0. 7 70 3 5\times {\text{B}}^{ 2} \times {\text{C}}} \right) + ( 1 4. 2 4000 \times {\text{B}} \times {\text{C}}^{ 2} ) \\ \end{aligned}$$

For MO:

5 $$\begin{aligned} {\text{Y}}^{0. 1} = \left( {\text{Peak Area}} \right)^{0. 1} & = - 1 4. 7 6 2 5 2+ \left( { 3. 7 8 3 9 1\times {\text{A}}} \right) + \left( {0.0 3 7 9 7 3\times {\text{B}}} \right) + \left( {0. 1 3 5 2 4\times {\text{C}}} \right) + \left( {0. 1 80 10 \times {\text{D}}} \right) \\ & \quad {-}\left( { 4. 60 6 4 7\times 10^{ - 4} \times {\text{A}} \times {\text{B}}} \right) + \left( { 3. 4 8 3 1 2\times 10^{ - 3} \times {\text{A}} \times {\text{C}}} \right){-}\left( { 6. 2 1 4 2 8\times 10^{ - 4} \times {\text{A}} \times {\text{D}}} \right){-}\left( { 1. 2 9 9 1 3\times 10^{ - 4} \times {\text{B}} \times {\text{C}}} \right) \\ & \quad + \left( { 1. 2 8 1 3 2\times 10^{ - 4} \times {\text{B}} \times {\text{D}}} \right){-}\left( { 5.0 2 1 2 7\times 10^{ - 4} \times {\text{C}} \times {\text{D}}} \right){-}\left( {0. 30 3 1 4\times {\text{A}}^{ 2} } \right){-}\left( { 5. 9 4 9 7 8 { 1}0^{ - 5} \times {\text{B}}^{ 2} } \right) \\ & \quad {-}\left( {0.0 1 1 1 4 2\times {\text{C}}^{ 2} } \right){-}\left( {0.0 20 9 1 8\times {\text{D}}^{ 2} } \right) \\ \end{aligned}$$

**Fig. 3:**
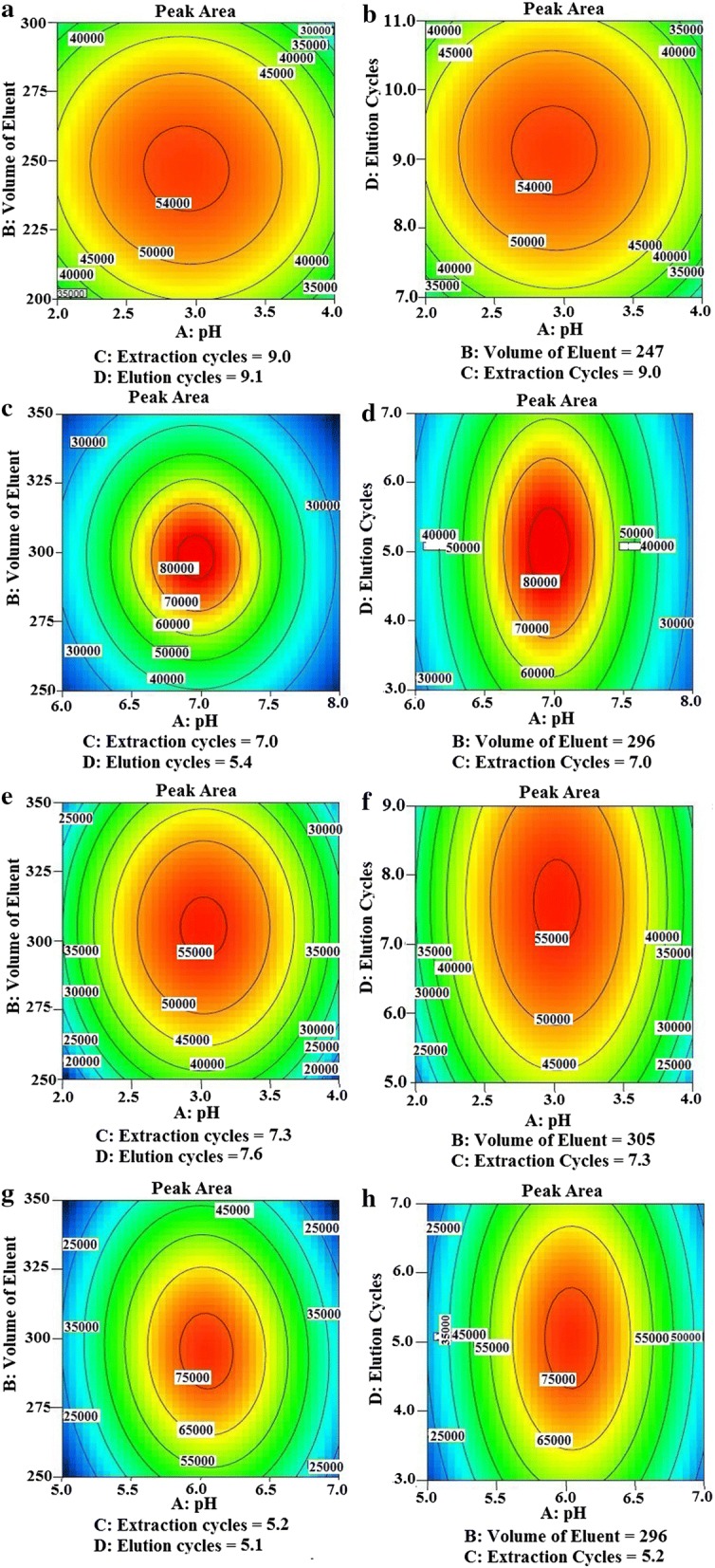
Response surface -2D contours showing the effect of independent variable on the extraction efficiency of dyes. **a** and **b** for MG, **c** and **d** for MO, **e** and **f** for RB and **g** and **h** for AR

By solving these equations for the condition of (∂Y/∂A) = 0, (∂Y/∂B) = 0, (∂Y/∂C) = 0, (∂Y/∂D) = 0, the critical point in the surface response can be achieved [33]. These critical points for this research are as follows: pH (A) = 3.02 for RB, 2.93 for MG, 6.04 for AR and 6.88 for MO, eluent volume (B) (µL) = 305 (for RB), 247 (for MG), 296 for AR and MO, the number of extraction cycles (C) = 7.3 for RB, 9 for MG, 5.2 for AR, and 7 for MO, the number of elution cycles (D) = 7.6 for RB, 9.1 for MG, 5.1 for AR, and 5.4 for MO. The ANOVA of regression of each model (indicated in Additional file 1: Table S2) demonstrates which model is of higher significance and with the determination coefficients (R^2^), the goodness-of-fit of each model can be checked. The value of adjusted R^2^ (0.946, 0.713, 0.885 and 0.956 for RB, MG, AR and MO, respectively) indicates that only 5.4% (RB), 28.7% (MG), 11.5% (AR) and 4.4% (MO) of the total variations were not explained with these models. In addition, good relation between the experimental and predicted values of the response was obtained, since the values of determination coefficient are close to unity (R^2^ = 0.969, 0.856, 0.942 and 0.978 for RB, MG, AR and MO, respectively). The quadratic model is statistically significant for the response, because the lack-of-fit is > 0.05. Moreover, based on what reported by Yetilmezsoy et al. [33], with low values of coefficient of variations (CV = 9.99, 37.30, 1.91, 8.41 for RB, MG, AR and MO, respectively), a high degree of precision and a good deal of the reliability of the conducted experiments is obtained. Based on the Fisher’s *F*-test results (*F*_model_ = 41.61, 5.96, 16.33 and 44.21 for RB, MG, AR and MO, respectively) and a very low probability value (*p*), the ANOVA of the regression models shows that quadratic models are also significant. In Fig. 3 two dimensional response surfaces as the function of other variable are shown.

### Analytical performance

#### Linear range, limit of detection and enrichment factor

The linearity of the proposed method was examined under the optimized conditions. Over a concentration range of 0.5–200.0 µg/L for RB and MG; and 1.0–50.0 µg/L for AR and MO, the calibration curve was linear. The least square equations over the dynamic linear range are indicated in Table 2. The limit of detection of the method for all target analytes was calculated using *3S*_*b*_/*m* equation (where *S*_*b*_ is the standard deviation of 7 consecutive measurements of the blank and *m* is shop of the calibration curve) and was 0.09, 0.17, 0.33 and 0.38 for RB, MG, AR and MO, respectively.

**Table 2:** Analytical figure of merit for MOF-PT-SPE combined by HPLC for determination of dyes (C and A are the concentrations of dyes and HPLC response as peak area, respectively)

Analyte	Linearity range (µg/L)	Equation of calibration	Determination coefficient (R^2^)	Limit of detection (µg/L)	Enrichment factor
RB	0.5–200.0	A = 225.7 C + 7435	0.9908	0.09	25.8
MG	0.5–200.0	A = 267.43 C + 1190.6	0.999	0.17	31.0
AR	1.0–150.0	A = 476.6 C + 6973.8	0.9953	0.33	25.8
MO	1.0–150.0	A = 467.95 C + 7909.7	0.9970	0.38	25.8

To achieve a high enrichment factor (EF), the effect of the sample volume on the recovery of dyes was investigated in the range of 2 to 10 for all of the analytes. The results showed that the extraction efficiency of selected dyes were very efficient (> 97%) in a sample volume of 8 mL and at the eluent solvent of 300 µL (for RB, MO and AR), 250 µL (for MG).

By mathematical calculation from the volume ratio of the sample to extracting phase, and a recovery of 97%, it is expected to have a pre-concentration factor of 26.6 (for RB, MO and AR), 32.0 (for MG). The real enrichment factors were experimentally achieved were 25.8 (for RB, MO and AR), 31.0 (for MG), and 28.2 (for AR). Table 3 compares the characteristic data of present method with those reported in the literature.

**Table 3:** Characteristic data of the suggested technique with other methods

Dye	Method	Detection method	LOD (µg/L)	Linear range (µg/L)	Refs.
Orang G, MO, AR	Micro-cloud point	Spectrophotometry	0.6–111.0	200–12,000	[8]
MG	MIP	HPLC	0.17	0–200	[14]
MG, RB and crystal violet	Micro-cloud point	Spectrophotometry	2.2	60–800	[17]
MG, gentian violet, leucomalachite and leucogentian	MIP	HPLC	0.11	10–250	[36]
RB, MG, MO, AR	MOF-PT-SPE	HPLC	0.09–0.38	0.5–200.0	This research

#### Determination of dyes in seawater samples

**Fig. 4:**
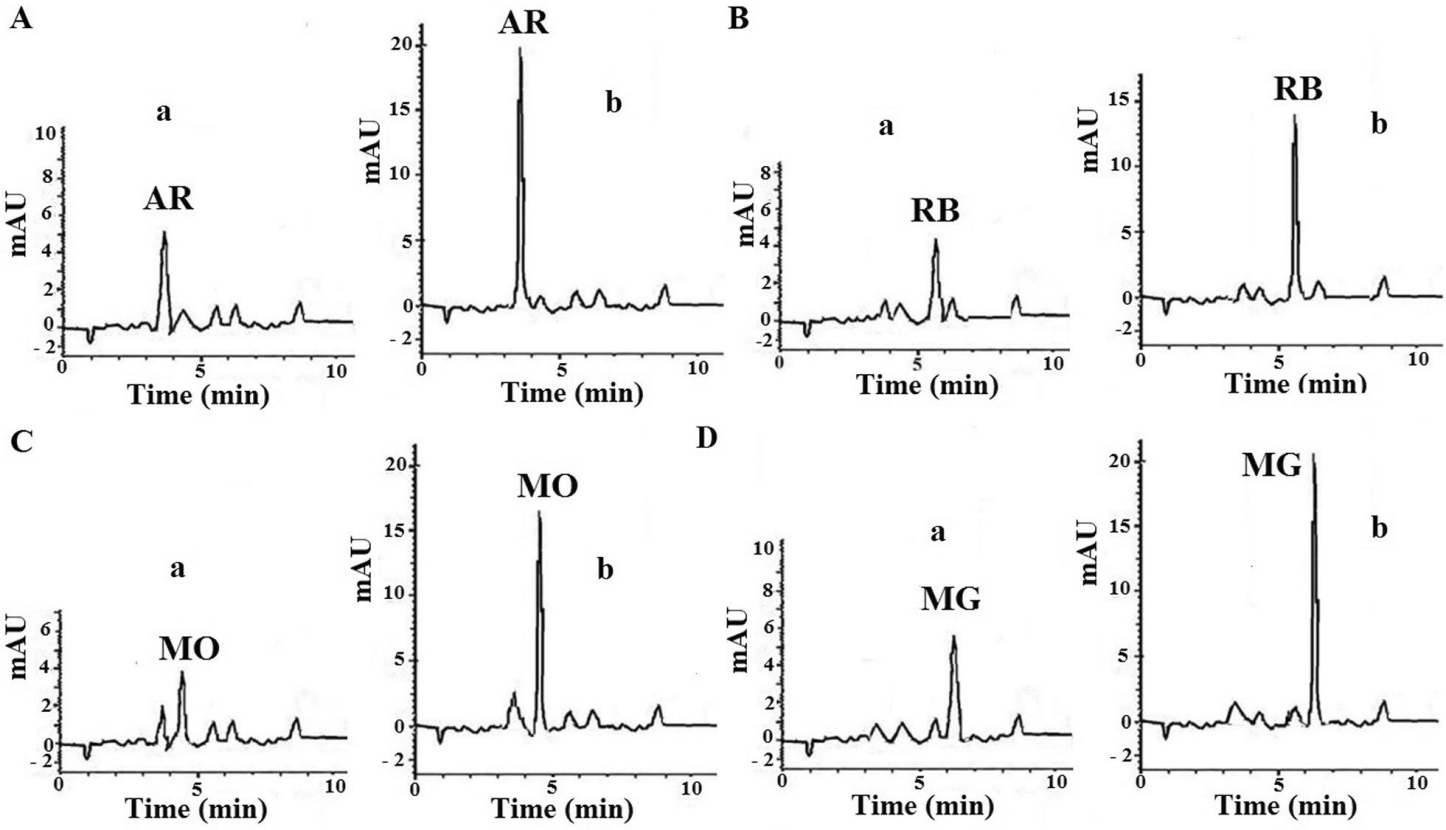
Sample HPLC chromatograms of sea water sample taken from station 3 (Chabahar Maritime University). Wavelengths of 510 nm for AR (**A**), 555 nm for RB (**B**), 448 nm for MO (**C**) and 618 nm for MG (**D**) were used. **a** MOF-PT-SPE without spiking, **b** MOF-PT-SPE of 10 µg/L spiked sample

The performance of proposed method was investigated by extraction and determination of dyes in five seawater samples taken from different spots of Oman Sea, close to Chabahar Bay (southern-east part of Iran). No salt was added to the real samples since they are fully salt saturated by themselves. Since no dyes could be detected in them, to evaluate the effect of sample media on recovery, they were spiked at the concentration of 10 µg/L with dyes. Results are presented in Table 4. Figure 4 shows sample chromatograms obtained for the analysis of seawater sample, taken from station 3. Significant raise of signal can be observed. Reproducibility of the method (as RSD%) was found to be in the range of 0.7–4.6% for RB, 0.6–4.0 for MG, 1.9–6.4 for MO and 0.7–6.3 for AR. These results show that the proposed technique can be used for determination of selected dyes in very complicated matrices such as seawater.

**Table 4:** Recovery results for real sample achieved from several points of Chabahar Bay (Iran)

Analyte added	Sampling location	Recovery % at spiked level of 10 (µg/L)	Dyes found (µg/L)	RSD (%)^b^
RB	Station 1, Tis^a^	–	1.56	0.7
	Station 1, Tis	94.4	11.00	2.6
	Station 2, Lypar^a^	–	1.67	3.4
	Station 2, Lypar	95.6	11.23	3.6
	Station 3, Chabahar Maritime University^a^	–	1.96	2.4
	Station 3, Chabahar Maritime University	93.6	11.32	4.6
	Station 4, Konarak^a^	–	1.44	1.9
	Station 4, Konarak	88.8	10.32	1.7
	Station 5, Kalantary^a^	–	2.77	1.3
	Station 5, Kalantary	91.1	11.88	2.6
MG	Station 1, Tis^a^	–	1.12	0.66
	Station 1, Tis	90.4	10.16	0.63
	Station 2, Lypar^a^	–	1.35	3.5
	Station 2, Lypar	96.8	11.03	4.0
	Station 3, Chabahar Maritime University^a^	–	1.65	3.9
	Station 3, Chabahar Maritime University	98.7	11.52	3.0
	Station 4, Konarak^a^	–	1.89	3.9
	Station 4, Konarak	96.5	11.54	2.8
	Station 5, Kalantary^a^	–	3.45	2.5
	Station 5, Kalantary	99.6	13.41	4.0
MO	Station 1, Tis^a^	–	1.20	4.5
	Station 1, Tis	78.3	9.03	1.9
	Station 2, Lypar^a^	–	1.14	5.3
	Station 2, Lypar	95.0	10.64	4.8
	Station 3, Chabahar Maritime University^a^	–	1.87	4.6
	Station 3, Chabahar Maritime University	86.8	10.55	2.5
	Station 4, Konarak^a^	–	1.26	3.4
	Station 4, Konarak	97.8	11.04	2.1
	Station 5, Kalantary^a^	–	3.02	3.1
	Station 5, Kalantary	96.4	12.66	6.4
AR	Station 1, Tis^a^	–	1.34	0.8
	Station 1, Tis	97.2	11.06	0.7
	Station 2, Lypar^a^	–	1.28	1.2
	Station 2, Lypar	97.3	11.01	1.0
	Station 3, Chabahar Maritime University^a^	–	1.98	2.8
	Station 3, Chabahar Maritime University	95.9	11.57	3.0
	Station 4, Konarak^a^	–	1.42	2.5
	Station 4, Konarak	93.0	10.72	2.3
	Station 5, Kalantary^a^	–	2.87	5.6
	Station 5, Kalantary	78.5	10.72	6.3

^a^No spiking

^b^RSD, relative standard deviation for three replicate measurement

## Conclusion

In this paper, the combination of pipette tip solid phase microextraction by means of a novel metal organic framework with HPLC was successfully used for the analysis of dyes in seawater. This technique has enough simplicity and sensitivity to be employed for routine analysis of dyes in such complicated media. An additional advantage of the suggested technique is its easy operation. Besides, the technique is feasible for high number of samples due to its short processing time.

## Additional file

**Additional file 1: Table S1.** Box–Behnken design observed and predicted values (*this table shows how close are the values obtained by real runs to what obtained by design of experiments for all of the analytes studied*). **Table S2.** ANOVA for preconcentration of dyes (*this table shows which model is of higher significance and what are the total variations which were not explained with these models*).

## Abbreviations

RSM: response surface methodology
MG: malachite green
RB: rhodamine B
MO: methyl orange
AR: acid red 18
LC–MS: liquid chromatography–mass spectrometry
LC–MS/MS: liquid chromatography–tandem mass spectrometry
GC–MS: gas chromatography–mass spectrometry
HPLC: high performance liquid chromatography
HPLC–MS: high performance liquid chromatography–mass spectrometry
LLE: liquid–liquid extraction
LPME: liquid phase microextraction
SPE: solid phase extraction
MIP: molecular imprinted polymer
CPE: cloud point extraction
MOFs: metal–organic framework
PT-SPE: pipette-tip solid phase extraction
CV: coefficient of variation
LOD: limit of detection
