# Supplementary material for: Application of response surface methodology for optimization of metal–organic framework based pipette-tip solid phase extraction of organic dyes from seawater and their determination with HPLC
Source: BMC Chem. 2019 Apr 23;13(1):59. doi: 10.1186/s13065-019-0572-0 (PMC6661774; doi:10.1186/s13065-019-0572-0)
Supplement: Supplementary file 1 — Additional file 1: Table S1. Box–Behnken design observed and predicted values (this table shows how close are the values obtained by real runs to what obtained by design of experiments for all of the analytes studied). Table S2. ANOVA for preconcentration of dyes (this table shows which model is of higher significance and what are the total variations which were not explained with these models). [file 13065_2019_572_MOESM1_ESM.docx]

**Additional data to:**

Application of response surface methodology for optimization of metal-organic framework based pipette-tip solid phase extraction of organic dyes from seawater and their determination with HPLC

Sayyed Hossein Hashemi^1^, Massoud Kaykhaii^2,*^[[1]](#footnote-1)^^, Ahmad Jamali Keikha^3^, Elahe Mirmoradzehi^1^ and Ghasem Sargazi^4^

^1^Department of Marine Chemistry, Faculty of Marine Science, Chabahar Maritime University, Chabahar, Iran. Email: sayyedhossein.hashemi62@gmail.com

^2^Department of Chemistry, Faculty of Sciences, University of Sistan and Baluchestan, Zahedan 98155-674, Iran. Email: kaykhaii@chem.usb.ac.ir; Tel: +98(54)33446413; Fax: +98(54)33431067

^3^Department of Mechanical Engineering, Faculty of Marine Engineering, Chabahar Maritime University, Chabahar, Iran. Email: a.j.keikha@cmu.ac.ir

^4^Department of Nano Chemistry, Graduate University of Advanced Technology, Kerman, Iran.

Email: g.sargazi@gmail.com

**Table S1 Box-Behnken design observed and predicted values**

| **A** | **B** | **C** | **D** | **Peak area obtained (MG)** | **Peak area predicted (MG)** | **Error% (MG)** | **Peak area obtained (MO)** | **Peak area predicted (MO)** | **Error% (MO)** | **Peak area obtained (RB)** | **Peak area predicted (RB)** | **Error% (RB)** | **Peak area obtained (AR)** | **Peak area predicted (AR)** | **Error% (AR)** |
| --- | --- | --- | --- | --- | --- | --- | --- | --- | --- | --- | --- | --- | --- | --- | --- |
| -1 | -1 | 0 | 0 | 29694 | 31786.97 | -7.05 | 22758 | 21488.2 | 5.58 | 16473 | 12524 | 23.97 | 17894 | 14631.07 | 18.23 |
| -1 | 0 | -1 | 0 | 35421 | 30864.84 | 12.86 | 30859 | 29658.2 | 3.89 | 21573 | 20486 | 5.04 | 22379 | 21531.04 | 3.79 |
| -1 | 0 | 0 | -1 | 34583 | 30031.52 | 13.16 | 23573 | 24670.8 | -4.66 | 17583 | 17706 | 0.70 | 15739 | 18071.9 | -14.82 |
| -1 | 0 | 0 | +1 | 38732 | 35240.91 | 9.01 | 25696 | 28241.6 | -9.91 | 25735 | 25807 | 0.28 | 17936 | 19112.9 | -6.56 |
| -1 | 0 | +1 | 0 | 37521 | 34258.88 | 8.69 | 32474 | 32018.8 | 1.40 | 23473 | 24087 | 2.62 | 23026 | 21452.12 | 6.84 |
| -1 | +1 | 0 | 0 | 29934 | 30677.69 | -2.48 | 21683 | 21202.5 | 2.22 | 10945 | 15133 | 38.26 | 12947 | 14420.38 | -11.38 |
| 0 | -1 | -1 | 0 | 34572 | 32545.93 | 5.86 | 34857 | 36694.3 | -5.27 | 25684 | 25677 | 0.03 | 32749 | 41181.4 | -25.75 |
| 0 | -1 | 0 | -1 | 33274 | 30231.46 | 9.14 | 29684 | 29241.0 | 1.49 | 24583 | 30627 | 24.59 | 38987 | 39358.52 | -0.95 |
| 0 | -1 | 0 | +1 | 40782 | 34427.51 | 15.58 | 36467 | 34254.6 | 6.07 | 29846 | 31623 | 5.95 | 34572 | 37599.02 | -8.76 |
| 0 | -1 | +1 | 0 | 36291 | 31975.82 | 11.89 | 35935 | 39767.4 | -10.66 | 31946 | 31937 | 0.03 | 36989 | 47067.47 | -27.25 |
| 0 | 0 | -1 | -1 | 24752 | 30155.34 | -21.83 | 38958 | 40704.0 | -4.48 | 34372 | 35037 | 1.93 | 55483 | 48034.71 | 13.42 |
| 0 | 0 | -1 | +1 | 28362 | 32337.42 | -14.02 | 65793 | 69170.1 | -5.13 | 42846 | 43138 | 0.68 | 58952 | 50800.66 | 13.83 |
| 0 | 0 | 0 | 0 | 54738 | 54835.44 | -0.18 | 71386 | 74783.8 | -4.76 | 55674 | 54877 | 1.43 | 74837 | 77240.75 | -3.21 |
| 0 | 0 | 0 | 0 | 56835 | 54835.44 | 3.52 | 74082 | 74783.8 | -0.95 | 53683 | 54877 | 2.22 | 78593 | 77240.75 | 1.72 |
| 0 | 0 | 0 | 0 | 52952 | 54835.44 | -3.56 | 72046 | 74783.8 | -3.80 | 56853 | 54877 | 3.48 | 75841 | 77240.75 | -1.85 |
| 0 | 0 | 0 | 0 | 57483 | 54835.44 | 4.61 | 79472 | 74783.8 | 5.90 | 52736 | 54877 | 4.06 | 76893 | 77240.75 | -0.45 |
| 0 | 0 | 0 | 0 | 51957 | 54835.44 | -5.54 | 77539 | 74783.8 | 3.55 | 55472 | 54877 | 1.07 | 80482 | 77240.75 | 4.03 |
| 0 | 0 | +1 | -1 | 28942 | 29477.21 | -1.85 | 60573 | 57053.0 | 5.81 | 43034 | 38638 | 10.22 | 57853 | 50878.96 | 12.05 |
| 0 | 0 | +1 | +1 | 33842 | 34711.12 | -2.57 | 63793 | 58640.0 | 8.08 | 44394 | 46739 | 5.28 | 59835 | 52354.98 | 12.50 |
| 0 | +1 | -1 | 0 | 28842 | 26524.2 | 8.04 | 32583 | 33862.3 | -3.93 | 28945 | 28938 | 0.02 | 37592 | 37908.63 | -0.84 |
| 0 | +1 | 0 | -1 | 26857 | 26258.92 | 2.23 | 28683 | 27286.1 | 4.87 | 27386 | 26131 | 4.58 | 31863 | 30181.44 | 5.28 |
| 0 | +1 | 0 | +1 | 34742 | 29841.9 | 14.10 | 35684 | 32186.1 | 9.80 | 46859 | 41337 | 11.78 | 33821 | 34541.35 | -2.13 |
| 0 | +1 | +1 | 0 | 33852 | 29309.25 | 13.42 | 33786 | 36773.8 | -8.84 | 45295 | 45286 | 0.02 | 35382 | 36220.25 | -2.37 |
| + 1 | -1 | 0 | 0 | 28583 | 29945.48 | -4.77 | 20483 | 20810.7 | -1.60 | 18435 | 14538 | 21.14 | 30752 | 20594.84 | 33.03 |
| + 1 | 0 | -1 | 0 | 32953 | 28466.86 | 13.61 | 29952 | 27393.6 | 8.54 | 22392 | 22500 | 0.48 | 21753 | 24101.18 | -10.79 |
| + 1 | 0 | 0 | -1 | 29436 | 26491.7 | 10.00 | 22683 | 22855.9 | -0.76 | 20943 | 19721 | 5.84 | 17532 | 21538.1 | -22.85 |
| + 1 | 0 | 0 | +1 | 31031 | 28880.8 | 6.93 | 24847 | 26114.7 | -5.10 | 26826 | 27821 | 3.71 | 19586 | 22345.19 | -14.09 |
| + 1 | 0 | +1 | 0 | 30261 | 26609.1 | 12.07 | 30957 | 28946.3 | 6.50 | 24692 | 26101 | 5.71 | 24783 | 26568.99 | -7.21 |
| + 1 | +1 | 0 | 0 | 19953 | 21139.94 | -5.95 | 18494 | 19323.1 | -4.48 | 14583 | 17147 | 17.58 | 16052 | 14344.41 | 10.64 |

**Table S2 ANOVA for preconcentration of dyes**

| **Source** | **Sum of**  **Squares** | **df** | **Mean**  **Square** | **F Value** | **p-value**  **Prob > F** | **% PC= (SS/ ∑ SS) × 100 [33]** |
| --- | --- | --- | --- | --- | --- | --- |
| **For MG** |  |  |  |  |  |  |
| Model | 1.89 × 10^19^ | 14 | 1.35 × 10^18^ | 5.96 | < 0.0001 |  |
| A-pH | 2.77 × 10^17^ | 1 | 2.77 × 10^17^ | 1.22 | < 0.0001 | 0.95 |
| B-Volume of Eluent | 2.02 × 10^17^ | 1 | 2.02 × 10^17^ | 0.89 | 0.51 | 0.69 |
| C-Extraction Cycles | 1.06 × 10^16^ | 1 | 1.06 × 10^16^ | 0.05 | 0.30 | 0.036 |
| D-Elution Cycles | 1.67 × 10^17^ | 1 | 1.67 × 10^17^ | 0.74 | 0.030 | 0.57 |
| AB | 3.62 × 10^16^ | 1 | 3.62 × 10^16^ | 0.16 | 0.044 | 0.12 |
| AC | 2.61 × 10^16^ | 1 | 2.61 × 10^16^ | 0.11 | 0.82 | 0.89 |
| AD | 1.08 × 10^16^ | 1 | 1.08 × 10^16^ | 0.05 | 0.0031 | 0.037 |
| BC | 9.24 × 10^15^ | 1 | 9.24 × 10^15^ | 0.04 | 0.22 | 0.032 |
| BD | 1.24 × 10^15^ | 1 | 1.24 × 10^15^ | 0.005 | 0.75 | 4.25 × 10^-3^ |
| CD | 9.96 × 10^15^ | 1 | 9.96 × 10^15^ | 0.04 | 0.40 | 3.41 × 10^-2^ |
| A^2 | 7.73 × 10^18^ | 1 | 7.73 × 10^18^ | 34.11 | 0.19 | 26.50 × 10 |
| B^2 | 7.73 × 10^18^ | 1 | 7.73 × 10^18^ | 34.12 | 0.47 | 26.50 × 10 |
| C^2 | 6.54 × 10^18^ | 1 | 6.54 × 10^18^ | 28.88 | 0.64 | 22.40 × 10 |
| D^2 | 6.43 × 10^18^ | 1 | 6.43 × 10^18^ | 28.37 | 0.76 | 22.00 × 10 |
| Residual | 3.17 × 10^18^ | 14 | 2.27 × 10^17^ |  | 0.84 |  |
| Lack of Fit | 2.9 × 10^18^ | 10 | 2.9 × 10^17^ | 4.23 | 0.53 |  |
| Pure Error | 2.74 × 10^17^ | 4 | 6.85 × 10^16^ |  | 0.72 |  |
| Cor Total | 2.21 × 10^19^ | 28 |  |  | 0.53 |  |
| **For MO** |  |  |  |  |  |  |
| Model | 3.92 × 10^-9^ | 14 | 2.8 × 10^-10^ | 44.21 | < 0.0001 |  |
| A-pH | 2.8 × 10^-11^ | 1 | 2.8 × 10^-11^ | 4.41 | 0.05 | 0.63 |
| B-Volume of Eluent | 1.4 × 10^-11^ | 1 | 1.4 × 10^-11^ | 2.21 | 0.16 | 0.313 |
| C-Extraction Cycles | 1.48 × 10^-11^ | 1 | 1.48 × 10^-11^ | 2.34 | 0.15 | 0.331 |
| D-Elution Cycles | 8.4 × 10^-11^ | 1 | 8.4 × 10^-11^ | 13.26 | 0.003 | 1.88 |
| AB | 2.36 × 10^-12^ | 1 | 2.36 × 10^-12^ | 0.37 | 0.55 | 0.528E-02 |
| AC | 6.96 × 10^-14^ | 1 | 6.96 × 10^-14^ | 0.011 | 0.92 | 1.56 × 10^-3^ |
| AD | 2.8 × 10^-14^ | 1 | 2.8 × 10^-14^ | 0.004 | 0.95 | 6.26 × 10^-4^ |
| BC | 1.35 × 10^-14^ | 1 | 1.35 × 10^-14^ | 0.002 | 0.96 | 3.02 × 10^-4^ |
| BD | 8.24 × 10^-14^ | 1 | 8.24 × 10^-14^ | 0.013 | 0.91 | 1.84 × 10^-3^ |
| CD | 2.32 × 10^-11^ | 1 | 2.32 × 10^-11^ | 3.66 | 0.08 | 0.519 |
| A^2 | 2.82 × 10^-9^ | 1 | 2.82 × 10^-9^ | 445.37 | < 0.0001 | 63.1 |
| B^2 | 1.3 × 10^-9^ | 1 | 1.3 × 10^-9^ | 204.67 | < 0.0001 | 29.1 |
| C^2 | 3.54 × 10^-13^ | 1 | 3.54 × 10^-13^ | 0.056 | 0.82 | 7.92 × 10^-3^ |
| D^2 | 1.8 × 10^-10^ | 1 | 1.8 × 10^-10^ | 28.35 | 0.0001 | 4.03 |
| Residual | 8.87 × 10^-11^ | 14 | 6.34 × 10^-12^ |  |  |  |
| Lack of Fit | 8.72 × 10^-11^ | 10 | 8.72 × 10^-12^ | 22.84 | 0.004 |  |
| Pure Error | 1.53 × 10^-12^ | 4 | 3.82 × 10^-13^ |  |  |  |
| Cor Total | 4.01 × 10^-9^ | 28 |  |  |  |  |
| **For RB** |  |  |  |  |  |  |
| Model | 5.27 × 10^9^ | 12 | 4.39 × 10^8^ | 41.61 | < 0.0001 |  |
| A-pH | 12178660 | 1 | 12178660 | 1.15 | 0.30 | 0.19 |
| B-Volume of Eluent | 13613762 | 1 | 13613762 | 1.29 | 0.27 | 0.21 |
| C-Extraction Cycles | 25956012 | 1 | 25956012 | 2.46 | 0.14 | 0.40 |
| D-Elution Cycles | 1.97 × 10^8^ | 1 | 1.97 × 10^8^ | 18.64 | 0.0005 | 3.02 |
| BC | 25441936 | 1 | 25441936 | 2.41 | 0.14 | 0.39 |
| BD | 50481025 | 1 | 50481025 | 4.78 | 0.04 | 0.77 |
| A^2 | 4.01 × 10^9^ | 1 | 4.01 × 10^9^ | 379.41 | < 0.0001 | 61.40 |
| B^2 | 1.5 × 10^9^ | 1 | 1.50 × 10^9^ | 141.68 | < 0.0001 | 23.00 |
| C^2 | 2.94 × 10^8^ | 1 | 2.94 × 10^8^ | 27.82 | < 0.0001 | 4.50 |
| D^2 | 3.42 × 10^8^ | 1 | 3.42 × 10^8^ | 32.37 | < 0.0001 | 5.24 |
| B^2C | 39562608 | 1 | 39562608 | 3.75 | 0.07 | 60.6 |
| BC^2 | 21629611 | 1 | 21629611 | 2.05 | 0.17 | 33.1 |
| Residual | 1.69 × 10^8^ | 16 | 10560376 |  |  |  |
| Lack of Fit | 1.58 × 10^8^ | 12 | 13171909 | 4.83 | 0.07 |  |
| Pure Error | 10903109 | 4 | 2725777 |  |  |  |
| Cor Total | 5.44 × 10^9^ | 28 |  |  |  |  |
| **For AR** |  |  |  |  |  |  |
| Model | 0.68 | 14 | 0.048 | 16.33 | < 0.0001 |  |
| A- pH | 0.006 | 1 | 0.0060 | 2.03 | 0.18 | 0.74 |
| B-Volume of Eluent | 0.007 | 1 | 0.0074 | 2.51 | 0.13 | 0.91 |
| C-Extraction Cycles | 0.0005 | 1 | 0.0005 | 0.17 | 0.69 | 0.06 |
| D-Elution Cycles | 0.0005 | 1 | 0.00047 | 0.16 | 0.70 | 0.06 |
| AB | 0.002 | 1 | 0.0021 | 0.72 | 0.41 | 0.26 |
| AC | 0.00019 | 1 | 0.0002 | 0.06 | 0.80 | 0.02 |
| AD | 6.18 × 10^-6^ | 1 | 6.18 × 10^-6^ | 0.002 | 0.96 | 0.0008 |
| BC | 0.00067 | 1 | 0.0007 | 0.23 | 0.64 | 0.083 |
| BD | 0.00066 | 1 | 0.0007 | 0.22 | 0.64 | 0.080 |
| CD | 1.61 × 10^-5^ | 1 | 1.61 × 10^-5^ | 0.005 | 0.94 | 0.0020 |
| A^2 | 0.60 | 1 | 0.60 | 201.25 | < 0.0001 | 73.05 |
| B^2 | 0.14 | 1 | 0.14 | 48.45 | < 0.0001 | 17.59 |
| C^2 | 0.013 | 1 | 0.01 | 4.35 | 0.06 | 1.58 |
| D^2 | 0.045 | 1 | 0.04 | 15.33 | 0.002 | 5.56 |
| Residual | 0.04 | 14 | 0.003 |  |  |  |
| Lack of Fit | 0.04 | 10 | 0.004 | 51.80 | 0.0009 |  |
| Pure Error | 0.0003 | 4 | 7.94 × 10^-5^ |  |  |  |
| Cor Total | 0.72 | 28 |  |  |  |  |

1. *Correspondence: kaykhaii@chem.usb.ac.ir

   ^2^Department of Chemistry, Faculty of Sciences, University of Sistan and Baluchestan, Zahedan 98155-674, Iran.

   Tel: +98(54)33446413, Fax: +98(54)33431067 [↑](#footnote-ref-1)
